# Cybersecurity Awareness Based on Software and E-mail Security with Statistical Analysis

**DOI:** 10.1155/2022/6775980

**Published:** 2022-03-18

**Authors:** Mohammed A. Alqahtani

**Affiliations:** Department of Computer Information Systems, College of Computer Science and Information Technology, Imam Abdulrahman Bin Faisal University, P.O. Box 1982, Dammam 31441, Saudi Arabia

## Abstract

The aim of this study is to discover the impact of software security and e-mail security on overall cybersecurity among the students of Imam Abdulrahman Bin Faisal University in Dammam. Another main purpose to conduct this study is to know the level of knowledge students have in the developing countries about the cybersecurity and how much are they mindful of cyber-attacks and the level of awareness among the university students. Two important hypotheses were studied to discover their importance in awareness of cybersecurity. One is software security, and the other is e-mail security. A total of 11 relevant questions were drafted, and then these questions were distributed among the university students, and around 390 responded to the questionnaires. Statistical analysis was performed on the responses using tools. Initial tests such as validity and reliability test, feasibility test of a variable, correlation test, multicollinearity test, multiple regression, and Heteroskedasticity test were conducted using SPSS. And furthermore, multiple linear regression model and coefficient of determination, hypothesis test, ANOVA test, and partial test were conducted using ANOVA. The outcome of the analysis is software security variable (X1) that has a significant and positive effect on cybersecurity awareness (*p* value ≤0.001, *β* = 0.192). This shows that having a thorough understanding of software security can raise cybersecurity awareness up to 19.2%. E-mail security variable (X2) has a significant and positive effect on cybersecurityawareness (*p*-value ≤0.000). This shows that having a thorough understanding of email security can raise cybersecurity awareness up to 31.3%. Software security (*X*1) and e-mail security (*X*2) variables simultaneously have a significant effect on cybersecurity awareness (*p*-value ≤0.000) with a correlation coefficient of 12.1% (*R*2 = 0.121). This shows that the independent variable used can explain the level of cybersecurity awareness up to 12.1%. Research results show that students are aware of software or application updates. Furthermore, students' awareness of email security is also good.

## 1. Introduction

The Internet is a worldwide computer network that has made it possible for individuals to quickly connect and exchange information. For almost every area, there is a vast quantity of knowledge available on the web. Direct contact, commercial transactions, and recreational purposes, such as web browsing, advertising campaigns, financial transactions, Internet shopping, are all examples of Internet applications. With all of the benefits that the Internet provides, it is also responsible for security and privacy problems. The online is the origin of all publicly available information that is being exploited, like accessing unfamiliar sites, Internet fraud, and unwittingly providing information to other parties. The validity of the channel via which the information is transferred is shrouded in mystery [1]. Spamming and phishing are serious concerns in network security because they aim to thieve money and personal information [1]. Every individual who has a connection to the Internet has a significant impact on a company's cyber security. An institution's Internet security is the sum of all vulnerable security breaches caused by staff behaviors and vulnerabilities. Carelessness, mistakes, sickness, mortality, insider threats, and media manipulation vulnerabilities are all elements of a company's human assault surface. Spyware is a common and successful form of social manipulation that exploits the human attack surface. Phishing is the deceptive practice of sending emails purporting to be from respectable organizations or persons to persuade recipients to disclose personal information such as login passwords or credit card information. As per Proofpoint Inc.'s 2019 State of the Phish survey, over 83 percent of all answering businesses confirmed phishing attempts in the preceding year [2]. Institutional defenses should theoretically prevent these assaults; however, a separate assessment of almost 32 million emails considered “secure” by conventional safety protocols and sent to business mailboxes revealed that around 467,000 included phishing Websites [3]. These security vulnerabilities pose a significant danger to organizational cyber security and demonstrate cyber-attacks on the person threat surface's potential to circumvent developed security outer walls. According to a Positive Innovations study, 93 percent of cyber-attacks try to capture confidential material, such as authorized login details, which may subsequently be utilized to create a hostile influence inside the organization or retransmit confidential material [4]. Because of their efficacy, phishing attempts are putting significant financial pressure on national economies. According to Forbes, phishing costs American firms $500 million in 2017 [5]. Correspondingly, the Australian Competition and Consumer Commission reportedly said that it had “obtained nearly 200,000 fraud cases with alleged damages exceeding [AUD] $340 million” [6]. In this part, I'll give you a fast rundown of current advancements in each component of this combo method, as well as the pros and downsides of reducing cyber security risk. Even though improved programming techniques continue to influence software security, the cyber security software industry has mostly focused on virus detection. This is an unusual circumstance because, while it is in everyone's interest in cyber security to prevent exploitable flaws, everyone believes that doing so is almost impossible for nontrivial programmes. Vendors have built little empires on this (so far) sound basis.

Because the majority of vulnerabilities are exploitable gaps generated by badly written code, the cyber security sector, in particular, has been vocal in its pursuit of solutions to improve the software. Unfortunately, most accuracy concerns have been masked by the push to modern DevOps development and software marketing requirements. Instead, the cyber security industry has mostly embraced a method for minimizing cyber security risk in software that consists of a hodgepodge of techniques, tools, and procedures, each of which targets a different aspect of the threat implications of bad code. In a 2013 research carried out by TNS Global for Halon, an e-mail security business, 30% of 1,000 people in the United States stated they would check email even if they knew it enclosed a virus or was doubtful [7]. Despite significant warnings about the hazards of opening unusual e-mails, the great most e-mail operators are nevertheless vulnerable to social engineering tactics [8]. To address the encounters raised by social engineering attacks, research-based endorsements give options to lessen the risk of a successful social engineering attack. Human or technical deployments can be used in social engineering attacks. Straightforward human contact is initiated by an offender who gets private information about a target and builds a connection with the person. Because the attacker uses a renowned or reputable party's approach, the target becomes susceptible and mistreated, and relinquishes personal or private company information, subsidizing to the jigsaw puzzle that the invader could use to his/her benefit. Technical attacks are less ambiguous and may be provided through several means, including software applications, e-mail attachments, and pop-up windows.

As the number of cybercrime attack techniques, kinds, and technologies aimed at exploiting individual vulnerabilities grows and changes, the relevance of the human aspect in cybercrime awareness and management grows. This indicates that, to fight cybercrime assaults meant to leverage human factors and preserve information assets, cyber security awareness campaigns that educate individuals on their roles and functions must be developed. According to surveys, one of several main reasons contributing to the increasing number of Internet-related assaults is a lack of knowledge of the danger of cyber security threats [9–11]. Students generally encourage data cracks and digital misbehavior due to their absence of understanding and consciousness of cyber security and the implications of cybercrime in research universities where a great lot of consumers are pupils. Moreover, university graduates have become major targets of hackers due to their frequent and growing accessibility to and exposure to numerous online apps and social platforms, which has escalated the cyber security threats connected with their everyday activities. Most students are unaware of the fundamental ideas of cyber security or the recommended procedures for protecting their computers from spyware, bugs, and frauds [12]. Pupils in Silicon Valley universities, for example (a tech-savvy atmosphere in the United States), showed lower levels of two-factor verification utilization or passcode sophistication for [13]accounts and even felt safe supplying personal information to an entire university citizenry notwithstanding knowing the risks [14]. According to cyber security assessment conducted in the United Kingdom, educational organizations have been the most prevalent victims in respect of recognized significant data theft or hacks in 2020 [15]. As a result, improving cyber security awareness at academic institutions in terms of understanding network security and protection methods has become critical. As a result, there is an urgent need to apply a standardized strategy to raising cyber security consciousness among college pupils, increasing their understanding of these concerns, and educating them on how to defend themselves from future intrusions.

The rest of the paper is organized as follows.  section 2 gives the literature review in this topic, section 3 describes the methodology followed to conduct this study, section 4 gives the results for all the tests conducted, and section 5 discusses all the test results acquired and the paper is concluded with a conclusion section followed by the appendix and references.

### 1.1. Literature Review

Cyber security consciousness for higher education students has been studied and continues to be studied to better understand pupils' attitudes, knowledge, behavior, and other pertinent influencing variables. During the Spring 2011 session, an information security study was administered to students in the College of Business and Economics at California State University, Los Angeles [16]. The primary issue with security awareness was revealed to be not a lack of security information, but rather how pupils utilize that knowledge in practical settings. There were recommendations to help institutions build courses that include more context-based cyber security awareness instruction. Another research revealed several critical aspects that determine consciousness and its link to other elements, like how spiritual signals and social conditioning affect peer behavior [17]. The proposal was that institutions of higher learning create rules and processes that encourage students to use appropriate answers to avert security problems. An assessment of students' cyber security awareness knowledge, mind-set, and behavior at seven Kuwaiti institutions revealed low levels and advised that formal cyber security awareness training be prioritized [18].

Two initial researches, one from Carnegie Mellon [19] and the second one from Berkeley [20], tried to uncover excellent forecasters of phishing pettiness. Both studies claimed that previous awareness of phishing and other types of Cybercrime was significant aspects in predicting a user's defencelessness to phishing attacks. Nevertheless, the small sample sizes (20 and 22, respectively) in each study decreased the significance of the findings. Downs, Holbrook, and Cranor presented the outcomes of a small-scale test run for larger-scale research that would be finished late that year. Their studies indicated (unconvincing) relations between specific demographics, prior phishing training, and phishing defencelessness [21]. The entire experiment was completed in 2010 by Sheng, Downs, and colleagues. They employed 1,001 assessment respondents online to respond to demographic questions and also several other technology-related queries [22]. Despite their outcomes being interesting, they were inadequate by the circumstance that respondents were more prone to be tech-savvy than the normal person because they were enrolled online. Recent research has shown that phishing offers a chimerical danger. An experiment done in 2015, with around 90 undergraduate psychology pupils, found no significant relationship between knowing if others marked an e-mail as phishing and actual phishing defencelessness [23]. As per a research done at the United States Coast Guard Academy, more than three-quarters of the 2017 new class got preyed to phishing by providing private data [24]. According to another analysis, their “serious phishing” website acquired 90% of login information [25]. Moody, Galletta, and Dunn examined 600 psychology and information systems pupils to determine whether there were any relationships between personality characters and phishing defenselessness, but they discovered few significant findings [26]. A study of 600 MBA pupils discovered that while phishing education was beneficial, phishing vulnerability remained high even after training [27]. Broadhurst et al. enrolled 140 students (the majority of whom were newsmen) and found no relations between demographic or scholastic features and phishing susceptibility [28].

Moreover, pupils from the University of New England's business department were questioned to evaluate their views about cyber security awareness, which will assist in the establishment of an effective information security awareness training program (ISAT). According to the survey findings, pupils recognize the value of ISAT in increasing their cyber security knowledge [29]. A study on security awareness was conducted among students and professionals in the Middle East academic sectors; the findings revealed a poor understanding of how to organize training in the best way to reduce cyber-attacks [30].

Several institutions are prone to cyber, which can be prompted by the stealing of patent prizes from both students and faculty, as well as the loss of faculty and students' records. This highlights the need of adding cyber security and training into any organization's security management strategy. Organizations must meet safety, confidentiality, trustworthiness, inspection, and regulatory criteria in addition to effective awareness programs, training, teaching, and policies.

Another emphasis of modern software security is deducing code security from the software process with which it is associated. That example, rather than reviewing software immediately for signs of malware or vulnerabilities, many security experts advocate investigating the product's development process. This is comparable to assessing a patient's health by asking about their behaviors rather than analyzing their blood. Simulator tools should also be provided to students, staff, and other experts to increase their degree of information safety training [31]. CyberCIEGE games were used to raise cyber security awareness for two Navy IT training programs, and preliminary findings indicate that the tools can be beneficial in raising cyber security awareness programs [32]. These examples show how technology and software may be used to increase student security awareness while also serving as a real-world experiment to help students understand the intricacies of security principles. Another study on cyber security awareness was performed at California State University's College of Business and Economics; the findings show that the basic problem with security awareness is not a lack of fundamental understanding, but instead, the method pupils use in everyday life; it also demonstrates that compliance with information security expertise is lesser than knowledge of it [16]. A cyber security awareness survey was conducted and assessed among Tamil Nadu college students (India). The participants were asked about numerous security dangers, and the poll found that 70 percent of 500 students are familiar with basic viral assaults and use virus protection, while 11 percent use obsolete antivirus and more than 97 percent do not realize where the infection came from [33]. The research was conducted in Malaysia to better understand the dangers of social networking site (SNS) frauds. The poll included 295 Malaysian students, and the results show that one-third of the students had been prey to SNS frauds [34]. This indicated that Malaysian undergraduate students are less conscious of cyber security issues on social media platforms. Another poll was conducted among 498 college students in the Pacific Northwest of the United States, and the findings indicated that the pupils could not define the terms malware (55 percent), Trojan horses (52 percent), phishing (50 percent), and worms (17 percent) [35]. Research on Internet activity and cyber security awareness was conducted among students of various ages in New Zealand, and the findings indicated that most students were unaware of fundamental cyber security terns and did not display an acceptable understanding of common phrases including phishing [36]. A cyber security awareness research was conducted among the population of Bangladesh, and the results suggest a patchy knowledge level that is insufficient [37]. This illustrates that many people are exposed to cyber-attacks, emphasizing the importance of the government implementing a cybersecurity awareness campaign. Many college students are aware of smartphone security issues, but they are also unaware of all security threats and proper security safeguards [38]. Some of the latest works on cybersecurity awareness are [39–42].

## 2. Materials and Methods

This study is based on a survey of 11 relevant questions drafted based on software and e-mail security to assess the awareness of cybersecurity among the students of Imam Abdulrahman Bin Faisal University (IAU). The purpose of this study is to assess cybersecurity awareness these two hypotheses were designed and each had a few questions that need to be answered by the students. These questions were distributed among the university students and around 390 students responded completely to all the questions. The students were of different capabilities; both male and female students participated in the survey. After receiving the responses. These students have different demographic gender, ages, education levels, and computer skills, and how often respondents make online purchases. These records were then applied for statistical analysis. The statistical analysis carried out on this dataset consisted of initial tests such as the validity and reliability test, the feasibility test of a variable, correlation test, multicollinearity test, multiple regression, and heteroskedasticity test were conducted using SPSS. And furthermore, multiple linear regression model and coefficient of determination, hypothesis test, ANOVA test, and partial test were conducted using ANOVA.

The questions in the software security use section were designed to elicit information about students' software updates behavior. The questions about the usage of security technologies were designed to assess current security practices among IAU University students. The e-mail security component was designed to evaluate students' comprehension of the security of the emails they often receive from unknown people. As a result, I investigated the students' cyber security awareness, abilities, behavior and attitudes, and self-perception. These questions are distributed among the undergraduate and postgraduate students and a total of 390 responses were received. Then these responses were again categorized based on the hypothesis and analysis. The following are the categories of the questions: questions based on software, e-mail, and activities. The responses to these questions were in multiple-choice answers with the following choices: strongly agree, agree, neutral, disagree, and strongly disagree.

The following are the questions drafted:

  Q1. My applications/software are automatically updated with no intervention from me
  Q2. My applications are manually updated by me when it is needed
  Q3. I do not update the applications/software of my device
  Q4. I usually reject any permission request from the application/software
  Q5. I usually open emails when received from an unknown sender
  Q6. Whenever I receive an e-mail asking about my details such as name, date of birth, age, and credit card number, I usually reply and send the required details
  Q7. I usually shop/purchase items advertised on my private e-mail
  Q8. I feel secure using a computer system and the Internet
  Q9. I think that my data on the university system\company system\personal PC are secure
  Q10. The password I use to access my bank account is more complex than the password used to access my social accounts
  Q11. I use the same password for both social networks such as Facebook, Twitter, iTunes, and my e-mail accounts

## 3. Results and Discussion

### 3.1. Demographic Data

The respondent profile was analyzed by obtaining data regarding gender, age, education level, computer skills, and how often respondents make online purchases. The sample size consisted of 390 participants representing the profile. The respondent profile data can be seen in Table 1.

Table 1 shows that the number of female respondents (54.1%) is higher than the number of male respondents (45.9%). According to the respondent's profile, the majority of the respondents are between the ages of 20 and 35, accounting for 55.1% of the total sample, with 39.6% of the sample being under the age of 20; the youth looked to be prominent. It is also worth noting that 4.9% of respondents said they were between the ages of 36 and 49 and only 0.5% of those who took part were between the ages of 50 and 65. When it comes to education, the bulk of the participants have a bachelor's degree, accounting for 92.6% of the total sample. Diploma students made up 3.%, master degree students made up 2.3%, and PhD students made up 1.5%. According to computer skills, the majority of respondents have intermediate computer skills (51.8%), followed by respondents with advanced computer skills (34.4%), and respondents with beginner computer skills (13.8%). When looking at online purchases, it is clear that the majority of the participants in this study bought things online regularly (70.5%), with only 29.5% buying things online occasionally. Summarizing the respondent profile, it can be noted that the respondents in this survey were largely filled by females, aged between 20 and 35 years old, with a bachelor's degree, intermediate computer skills, and a habit of making purchases online.

### 3.2. Description of the Variables

#### 3.2.1. Software Security (*X*_1_)

It is necessary to update software or application on a computer or mobile phone; software updates are vital because they frequently include critical security patches. Skipping software updates is a mistake that allows hackers to gain access to your personal information, placing you at risk of identity theft, financial loss, and more. In addition to security fixes, software updates might contain new or enhanced features, as well as improved interoperability with other devices or programs. They can also make your program more stable by removing old functionality [43]. Researchers wanted to determine the level of software security in students, and the following questions were posed to the group.

  Q1. My applications/software are automatically updated with no intervention from me
  Q2. My applications are manually updated by me when it is needed
  Q3. I do not update the applications/software of my device
  Q4. I usually reject any permission request from the application/software

**Figure 1:**
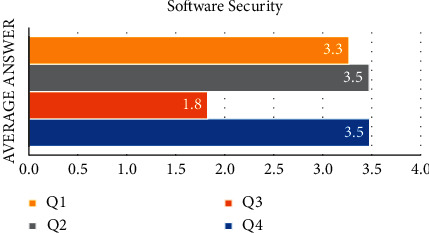
The average answer of each question in software security variable.

Figure 1 shows that the software security variable is made up of four statements. The average value of the responses to the first statement (Q1) is 3.3. This demonstrates that the majority of respondents chose “neutral” to “agree” to the provided statement. The average value of the respondent's answer for the second statement (Q2) and statement 4 (Q4) is 3.5. This demonstrates that the majority of respondents chose “neutral” to “agree” to the provided statement. And then, the third statement (Q3) received an average answer of 1.8, this demonstrates that the majority of respondents chose “disagree” in response to the provided statement. The results suggest that the majority of students are aware of the importance of email security. e-mail security (*X*_2_)

E-mail is used for a variety of purposes, including connecting with people, obtaining information, and applying for employment, internships, and scholarships. The formality, target audience, and desired effects of the messages you send will vary depending on your goals [44]. Malware, spam, and phishing assaults from unknown senders are frequently sent by e-mail. As a result, it is important to understand e-mail security so that data and information can be safeguarded. Researchers wanted to determine the level of email security in students, and the following questions were posed to the group.

  Q1. I usually open emails when received from an unknown sender
  Q2. Whenever I receive an e-mail asking about my details such as name, date of birth, age, and credit card number, I usually reply and send the required details
  Q3. I usually shop/purchase items advertised on my private e-mail

**Figure 2:**
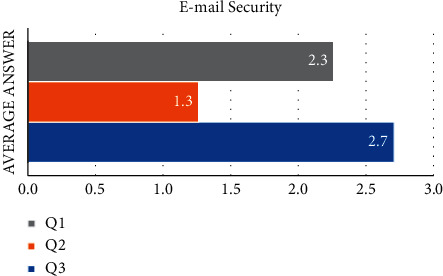
The average answer of each question in the email security variable.

Figure 2 shows that the email security variable is made up of three statements. The average value of the responses to the first statement (Q1) is 2.3. This demonstrates that the majority of respondents chose “disagree” with the provided statement. The average value of the respondent's answer for the second statement (Q2) is 1.3. This demonstrates that the majority of respondents chose “strongly disagree” with the provided statement. And the third statement (Q3) received an average answer of 2.7. This demonstrates that the majority of respondents chose “disagree” to “neutral” in response to the provided statement. The results suggest that the majority of students are aware of the importance of software updates.

#### 3.2.2. Cybersecurity Awareness (*Y*)

Almost all activities are now conducted online; nevertheless, in addition to enabling activities, the Internet is prone to cyber-attacks, which can expose our data and information to untrustworthy parties. As a result, understanding cybersecurity awareness is critical. Researchers wanted to determine the level of cybersecurity awareness in students, and the following questions were posed to the group.

  Q1. I feel secure using a computer system and the Internet
  Q2. I think that my data on the university system\company system\personal PC are secure
  Q3. The password I use to access my bank account is more complex than the password used to access my social accounts.
  Q4. I use the same password for both social networks such as Facebook, Twitter, iTunes, and my e-mail accounts.

**Figure 3:**
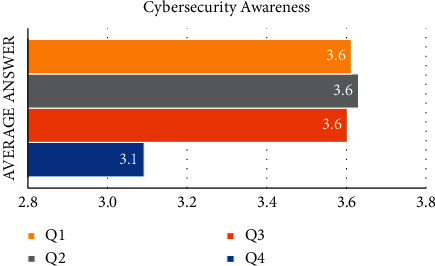
The average answer of each question in the cybersecurity awareness variable.

Figure 3 shows that the email security variable is made up of four statements. The average value of the responses to the first statement (Q1), the second statement (Q2), and the third statement (Q3) is 3.6. This demonstrates that the majority of respondents chose “agree” to the provided statement. The average value of the respondent's answer to the fourth statement (Q4) is 3.1. This demonstrates that the majority of respondents chose “neutral” to the provided statement.

### 3.3. Data Analysis

In this section, various statistical analysis techniques are discussed. The results of these techniques are displayed in tabular format. The various analyses are as follows: validity and reliability test, feasibility test of questions, correlation test between Variables, and assumption test.

### 3.4. Validity and Reliability Test

This research uses the correlation test (*r*) to assess the validity of the research question.  Table 2 shows the validity test results for each item from the 390 respondents. The validity coefficient (*r*-value) will be compared to its reference criterion, which takes the value from the *r* table.

Table 2 shows the findings of the validity assessment demonstrates that all questions from the independent variables, software security (*X*_1_) and email security (*X*_2_), and the dependent variable cybersecurity awareness (*Y*), have correlation coefficients (*r*-value) larger than r-table (0.115). This shows that each question is valid; therefore, it can be stated that all of the questions utilized in this study are appropriate for future research.

Following the validity test, the reliability test is used to determine the consistency of people's responses across numerous items on a multiple-item measure. The Cronbach's alpha was utilized as a measure of consistency in the reliability test, as indicated in Table 3.

Table 3 shows Cronbach's alpha value of 0.528. The Cronbach's alpha from the result ranges from 0.5 to 0.6; this demonstrates that each item in the research is reliable enough. In addition, future studies should include more items per variable and address the used items differently to obtain a more reliable scale. As a result, all of the statement items utilized in this study are suited for use in future research but adding more variables as predictor variables and adding more items per variable will be required to increase the reliability of results.

### 3.5. Feasibility Test of a Questions

The correlation between questions item was examined using Bartlett's test and the Kaiser –Meyer*–*Olkin (KMO) test. This test is used to determine the feasibility of questions that has been subjected to factor analysis.

Table 4 shows that Bartlett's test of sphericity has a significant value (*p* value) of 0.000, which is less than 0.05. It shows that there is a relation between the questions item. The Kaiser–Meyer–Olkin (KMO) value is 0.637; the KMO value is between 0.5 and 1 indicating that the questions are homogeneous. Both tests were passed, allowing for the anticipation of questions and subsequent study.

### 3.6. Correlation Test between Variables

Pearson correlation was used as a correlation test in this study to determine the level of relationship closeness between the dependent variable and the independent variable. The criteria for the strength of the correlation between the two variables are shown in Table 5 (Sarwono, 2006).

The correlation component matrix, shown in Table 6, contains the correlation values between the variables used in the study. The primary goal of this test is to determine the relationship between each independent variable, namely, software security (*X*_1_) and email security (*X*_2_) to the dependent variable, namely, cybersecurity awareness (*Y*).

Software security is positively related to cybersecurity awareness (*p* ≤ 0.000). The correlation value between software security and cybersecurity awareness is between 0.25 and 0.5, indicating a moderate level of relationship between software security and cybersecurity awareness (*r* = 0.243). E-mail security is positively related to cybersecurity awareness (*p* ≤ 0.000), with the correlation value ranging from 0.25 to 0.5 indicating a moderate level of relationship between e-mail security and cybersecurity awareness (*r* = 0.312). Both variables have a relationship with cybersecurity awareness; however, the relationship is modest to low in intensity.

### 3.7. Multiple Regression

Multiple regression is a form of linear regression that is more composite than normal linear regression. When we wish to estimate the worth of a variable centered on the values of two or more other variables, we utilize it. The variable we wish to estimate is referred to as the dependent variable (or sometimes, the outcome, target, or criterion variable). Multiple regression is a linear regression model extension that permits the forecasting of networks with multiple independent variables. This is accomplished by simply adding extra terms to the linear regression equation, every term signifying the impact of a discrete physical parameter.

### 3.8. Assumption Test

All parametric tests in statistical analysis make some hypothesis about the information, commonly known as assumptions. Breach of such assumptions alters the research's conclusion and interpretation of the results. The basic principle is as follows: if everything else is equal and A has a higher severity than B, then A is tested first. The second component is the likelihood of a hypothesis is correct. Many people find it paradoxical that hypotheses with a smaller chance of becoming correct must be examined first.

### 3.9. Normality Test

The normality assumption test is used to determine if the residuals or errors of the model are normally distributed. The following is the hypothesis for the normality test:

  H_0_: residual or error normally distributed
  H_1_: residual or error not normally distributed

If the significant value (*p*-value) is less than 0.05, then H_0_ is rejected, indicating that the residual is not normally distributed. If the significant value (*p*-value) is greater than 0.05, H_0_ is accepted, and the residual is normally distributed. The Kolmogorov–Smirnov test was performed to determine the normality test, and the results are reported in Table 7.

Table 7 shows the results of normality testing using the Kolmogorov–Smirnov statistical test. The *p-value* = 0.200 is greater than 0.05, indicating that *H*_0_ is accepted. This indicates that the residuals or errors are normally distributed, and the normality assumption has been met.

### 3.10. Multicollinearity Test

The multicollinearity test aims to examine a strong correlation between the independent variables. If the variance value of the inflation factor (VIF) is greater than 10 and the tolerance value is less than 0.10, the model has multicollinearity issues. If the VIF value is less than 10 and the tolerance value is greater than 0.10, the model is free of multicollinearity issues.  Table 8 demonstrates that the VIF values of the two variables are all less than 10 and the tolerance values are more than 0.10, indicating that none of the independent variables in this study experience multicollinearity issues.

### 3.11. Heteroskedasticity Test

The Heteroskedasticity test is used to evaluate if there is an inequality of variance from another observation in a regression model. The Glejser test is used in this heteroscedasticity test. If the significant value (*p*-value) is less than 0.05, the residual variance is considered heterogeneous. If the *p*-value is more than 0.05, the residual variance is homogeneous, and the heteroscedasticity criterion is met.  Table 9 shows the results of the heteroscedasticity test.

The results of the Glejser test are shown in (Table 9.). The *p*-value for each independent variable is greater than 0.05; this means that the regression model has no heteroscedasticity issues. The residual variance is homogeneous, and the heteroscedasticity assumption is met.

### 3.12. Multiple Linear Regression Model and Coefficient of Determination (*R*^2^)

Regression analysis was utilized to determine the relationship between the independent and dependent variables by providing a thorough picture of both (Calonico, 2019). After passing all of the assumption tests, the next step is to create multiple linear regression model equations. The developed regression model equation can be used to investigate the impact of software security and email security on cybersecurity awareness. The following is the multiple linear regression model derived from the regression coefficients in Table 10: The cybersecurity awareness is equated in equation (1) wherein *X*_1_ and *X*_2_ are hypothesis.

(1) $$\begin{array}{c} \text{Cybersecurity Awareness}=9.635+\;\left(0.196\right){\text{X}}_{1}+\left(0.313\right){\text{X}}_{2}\text{.} \end{array}$$

The magnitude of the regression coefficient above can be interpreted as follows: if student understanding of software security increases by 1%, cybersecurity awareness will improve by 19.6%. If student knowledge of email security increases by 1%, cybersecurity awareness will improve by 31.3%.

The coefficient of determination is a value that indicates how well the independent variable in the model can explain the variance in the dependent variable. Based on Table 11, the coefficient of determination (*R*^2^) is 0.121, which means the software and email security contribute 12.1% to the influence of cybersecurity awareness. While the residual value is 88.1% (100%–12.1%), it implies that there are additional factors that influence cybersecurity awareness that are not taken into this research.

### 3.13. Hypothesis Test

A statistical hypothesis method is a statistical reasoning procedure that is used to discover a probable result using two diverse and presumably contradictory, hypotheses. For the probability distribution of the data, a null hypothesis and an alternative hypothesis are provided in a statistical hypothesis test.

### 3.14. ANOVA Test (*F*-Test)

#### 3.14.1. ANOVA (Analysis of Variance)

ANOVA tests and evaluates the differences in the means of various sets, commonly used similarly as a *t*-test, but for more than two groups. Mood's median is a method for comparing the medians of two or more sample populations. Welch's *T*-test compares the means of two population samples. There are two kinds of hypotheses: null hypotheses and alternative hypotheses. A research project usually begins with a problem. Following that, these hypotheses give explicit restatements and explanations of the study topic to the scholar. The hypothesis is clear.

The following is an *F*-test to see whether the independent variable has a simultaneous effect on the dependent variable. The hypothesis in this test is as follows:

  H0: software security (*X*1) and e-mail security (*X*2) together are not significantly related to cybersecurity awareness (*Y*)
  H1: software security (*X*1) and e-mail security (*X*2) together are significantly related to cybersecurity awareness (*Y*)

Table 12 shows the results of the *p*-value (0.000) smaller than 0.05, indicating that software security (*X*_1_) and e-mail security (*X*_2_) have a significant effect on cybersecurity awareness when used together (Y).

### 3.15. Partial Test (*t*-Test)

To determine the significant influence of each independent variable on the dependent variable, the *t*-test is utilized. The partial test hypothesis is as follows:

  Hypothesis 1.
  H_0_: software security (*X*_1_) has no significant effect on cybersecurity awareness (*Y*)
  H_1_: software security (*X*_1_) has a significant effect on cybersecurity awareness (*Y*)
  Hypothesis 2.
  H_0_: e-mail security (*X*_2_) has no significant effect on cybersecurity awareness (*Y*)
  H_1_: e-mail security (*X*_2_) has a significant effect on cybersecurity awareness (*Y*)

Table 13 shows the results of partial hypothesis testing (*t*-test), based on these results, the conclusions are:

1. The software security variable (*X*_1_) has a *p-*value (0.0001) < (0.05), so it can be concluded that software security (*X*_1_) has a significant effect on sybersecurity swareness (*Y*)
2. The e-mail security variable (*X*_2_) has a *p-*value (0.0001) < (0.05), so it can be concluded that e-mail security (*X*_2_) has a significant effect on cybersecurity awareness (*Y*)

## 4. Discussion

Research results show that students are aware of software or application updates, as evidenced by the fact that most of the students in this study always update their software/application either manually or automatically with no intervention, and that most of them also reject any permission request from the application, such as asking for location and so on. Furthermore, students' awareness of email security is also good, as seen by respondents' responses to questionnaire questions, which reveal that most students do not reply e-mails from unknown senders. Most students strongly disagree in replying to personal information such as credit card numbers that are requested in email. Most students also rarely shop online using personal e-mail addresses.

According to cybersecurity awareness, the majority of students feel safe when using computers and the Internet, they do not encounter any suspicious activities that could compromise their personal information. The majority of them also believe that their personal information on the university system, company system, and home computer is safe. According to their password knowledge, the majority of them use a more complex password to access their bank accounts than they do for social media. Nonetheless, the majority of them continue to use the same password for social media. In this result, we can see that the majority of students are aware of and have prior knowledge of software security and email security, but that awareness of other cybersecurity issues is not high.

The *t*-test results suggest that the software security variable has a significant effect on cybersecurity awareness (*p* < 0.05). In the multiple linear regression model (Table 10), the regression coefficient of the password security variable has a beta value of 0.196. As a result, software security can be inferred to have a positive and significant impact on cybersecurity knowledge. The positive effect indicates that knowing software security will raise cybersecurity awareness by 19.6%. The email security variable also has significantly influenced cybersecurity awareness (*p* < 0.05). The email security variable has a beta value of 0.313 in the regression coefficient. As a result, e-mail security can be inferred to have a positive and significant impact on cybersecurity knowledge. It is indicated that knowing email security increases cybersecurity awareness by 31.3%, according to the beneficial effect.

The findings of simultaneous hypothesis testing using the *F*-test (Table 12) reveal that software and email security have substantial effects on cybersecurity awareness at the same time (*p* < 0.05). The strength of the influence provided by the two variables may be understood by looking at the coefficient of determination (*R*^2^), which is 0.121, indicating that software and email security contribute 12.1% towards the cybersecurity awareness among the students. Meanwhile, the remaining percentage of 87.9% implies that some other factors or variables influence cybersecurity awareness that is not considered as part of the variable in this research. The low *R*^2^ value shows that the two predictors variables utilized in this study have a minor impact on the level of cybersecurity awareness among students and adding more variables is necessary to boost the *R*^2^ value.

Because of the advancement of more advanced technology, the problem of cybersecurity awareness has been brought up many times. In [45], a general lack of understanding of cyber risk, which extended to app usage and content delivery on social media and online pages. In [12], the authors researched for students at Majmaah University, Saudi Arabia, stating that students at Majmaah University already have a high level of awareness of cybersecurity awareness (*R*^*2*^ *=* *0.55*). On the other hand, according to Haddlington (2018), large corporations have more vital cybersecurity awareness. The reason for this is that a large corporation has a cybersecurity policy in place and appropriate budgetary resources. This is because a large corporation has a cybersecurity policy in place as well as appropriate budgetary resources. In the case of students, if educational and academic institutions want to improve students' cybersecurity knowledge, they need to establish a specific training program [46].

## 5. Conclusions

In this study, awareness of cyber security was assessed on the students of Imam Abdulrahman Bin Faisal University. Two important hypotheses were designed to calculate their impact on the analysis. Software security and e-mail security were studied. The questions designed were based on these hypotheses. Then these questions were distributed among the students of the university with various demographic details. The responses received were 390 both from males and females. Then, statistical analysis was performed wherein various tests were conducted and finally based on the research findings described in the previous sections, the following summary is obtained: software security variable (*X*_1_) has a significant and positive effect on cybersecurity awareness (*p*-value ≤0.001, *β* = 0.192). This shows that having a thorough understanding of software security can raise cybersecurity awareness by up to 19.2%. The students in this study are aware of software security, they already pay attention to updating the software or application manually or automatically. E-mail security variable (*X*_2_) has a significant and positive effect on cybersecurity awareness (*p-value* ≤0.000, = 0.313). This shows that having a thorough understanding of email security can raise cybersecurity awareness by up to 31.3%. The students in this study are aware of email security and already take precautions such as not responding to unknown senders and rejecting any account that requests personal information. Software security (*X*_1_) and e-mail security (*X*_2_) variables simultaneously have a significant effect on cybersecurity awareness (*p*-value ≤ 0.000) with a correlation coefficient of 12.1% (*R*^2^ = 0.121). This shows that the independent variable used can explain the level of cybersecurity awareness up to 12.1%.

## 6. Future Scope and Research Directions

The future scope would be to work on real questions which are designed by the cybersecurity experts. The researchers can explore further to detect the real problems and figure out relevant questions to enhance the quality of the study.

## 7. Recommendations

The author would recommend helping institutions build courses which include more context-based cyber security awareness instruction. Also, students should educate themselves by attending webinars in cybersecurity awareness and get the latest information about the attacks.

## Figures and Tables

**Table 1:** Data demographic of the respondent in this research (*n* = 390).

Variable	Category	Number	Percentage (%)
*Gender*	Male	211	54.1
	Female	179	45.9

*Age*	<20	154	39.5
	20–35	215	55.1
	36–49	19	4.9
	50–65	2	0.5

*Education*	Diploma	14	3.6
	Bachelor degree	361	92.6
	Master degree	9	2.3
	PhD	6	1.5

*Computer skill*	Beginner	54	13.8
	Intermediate	202	51.8
	Advance	134	34.4

*Purchase online*	Rarely	115	29.5
	Frequently	275	70.5

**Table 2:** Validity test results.

Variable	Question's item	*r*-value	*r*-table
Software security (*X*_1_)	Q1	0.229	0.115
	Q2	0.506	
	Q3	0.625	
	Q4	0.616	
E-mail security (*X*_2_)	Q5	0.764	
	Q6	0.651	
	Q7	0.743	
Cybersecurity awareness (Y)	Q8	0.643	
	Q9	0.716	
	Q10	0.439	
	Q11	0.555	

**Table 3:** Reliability test results.

Cronbach's alpha	Number of items	Description
0.528	11	Reliable enough

**Table 4:** KMO dan Bartlett's Test.

Kaiser–Meyer–Olkin measure of sampling adequacy		0.637
Bartlett's test of sphericity	Approx. chi-square	585.703
	Df	55
	Sig.	0.000

**Table 5:** Guidelines for providing an interpretation of correlation coefficients.

Correlation value (r)	Interpretation
0	No correlation
>0–0.25	Low correlation
>0.25–0.5	Moderate correlation
>0.5–0.75	High correlation
>0.75–0.99	Very high correlation
1	Perfect correlation

**Table 6:** Correlation component matrix.

Variable	Software security	E-mail security	Cybersecurity awareness
Software security	1		
E-mail security	0.315 (*p* ≤ 0.000)	1	
Cybersecurity awareness	0.243 (*p* ≤ 0.000)	0.312 (*p* ≤ 0.000)	1

**Table 7:** Normality test result.

Uji Statistik	*p*-value
N	390
Kolmogorov–Smirnov	0, 200

**Table 8:** Multicollinearity test.

Variable	Tolerance	VIF value
Software security (*X*_1_)	0.901	1.110
E-mail security (*X*_2_)	0.901	1.110

**Table 9:** Heteroskedastisitas.

Variable	*p*-value
Software security (X1)	0, 261
E-mail security (X2)	0, 095

**Table 10:** Multiple linear regression coefficient.

Variable	Regression coefficient (*β*)
Intercept	9.632
Software security (*X*_1_)	0.196
E-mail security (*X*_2_)	0.313

**Table 11:** Correlation coefficient and determination.

Model	*R*	*R* ^2^	Adjusted *R*^2^
Regression	0.348	0.121	0.116

**Table 12:** *F*-test results.

Model	*F*	Sig (*p-value)*
Regression	26.589	0.000

a. Dependent variable: Cybersecurity awareness. 1.1 predictors: constant, software security, and e-mail security

**Table 13:** Result of *t*-test.

Variable	t-value	Sig (*p-value*)
Password security (*X*_1_)	3.209	0.001
Social media activities (*X*_3_)	5.207	0.000

## Data Availability

The data used to do this research was a questionnaire designed particularly for this study and was distributed among the students, and then analysis was carried out on that data. The data are in the form of an Excel file. The description about the dataset is discussed in the Materials and Methods Section of this paper.
